# Interactions between *Shigella flexneri* and the Autophagy Machinery

**DOI:** 10.3389/fcimb.2016.00017

**Published:** 2016-02-10

**Authors:** Sina Krokowski, Serge Mostowy

**Affiliations:** Department of Medicine, MRC Centre of Molecular Bacteriology and Infection, Imperial College LondonLondon, UK

**Keywords:** autophagy, cytoskeleton, innate immunity, inflammation, *Shigella flexneri*

## Abstract

Autophagy, an intracellular degradation process, is increasingly recognized as having important roles in host defense. Interactions between *Shigella flexneri* and the autophagy machinery were first discovered in 2005. Since then, work has shown that multiple autophagy pathways are triggered by *S. flexneri*, and autophagic responses can have different roles during *Shigella* infection. Here, we review the interactions between *S. flexneri* and the autophagy machinery, highlighting that studies using *Shigella* can reveal the breadth of autophagic responses available to the host.

## Introduction

The process of autophagy degrades intracellular material via fusion with lysosomes, and is evolutionarily conserved (de Duve and Wattiaux, 1966; Mizushima et al., 1998; Kabeya et al., 2000; Yang and Klionsky, 2010). It was originally discovered as a non-selective nutrient recycling process in response to starvation (Takeshige et al., 1992). More recently, it has been demonstrated that autophagy can act in a selective manner to maintain cellular homeostasis (Khaminets et al., 2015), and that selective autophagy plays an important role in innate immunity by eliminating intracellular pathogens (Levine et al., 2011; Shibutani et al., 2015). Studies using cellular (*in vitro*) and animal (*in vivo*) models have shown that a variety of intracellular bacterial pathogens can interact with the autophagy machinery, and autophagic responses can restrict or promote bacterial replication depending on the infection context. In this review, we discuss canonical and non-canonical autophagy pathways, their interplay with invasive bacteria, and highlight what has been discovered from their interactions with *Shigella flexneri*.

### Canonical and non-canonical autophagy pathways

The process of canonical autophagy is dependent on the systematic recruitment of about 40 autophagy-related (ATG) proteins to an isolation membrane called a phagophore (Feng et al., 2014). First, the Unc-51 like kinase (ULK) complex (ULK1, ULK2, ATG13, ATG101, FIP200), and ATG9L recruit the autophagy-specific class III phosphoinositide 3-kinase [PI(3)K] complex (ATG14L, VPS34, Beclin-1, VPS15) to generate phosphatidylinositol 3-phosphate [PI(3)P] required for phagophore formation. Next, members of the WD-repeat domain phosphoinositide-interacting (WIPI) protein family (WIPI1-WIPI4) bind PI(3)P, ATG2, and ATG16L1, and recruit the ATG16L1 complex (ATG16L1, ATG5, ATG12) to elongate the phagophore. Finally, the accumulation of ATG16L1 initiates the conjugation of microtubule-associated protein light chain 3 (LC3) family members to the phagophore, and closes the autophagosome. Thereafter, autophagosomes mature along the endocytic pathway and fuse with lysosomes to form degradative autolysosomes.

In contrast to canonical autophagy, non-canonical autophagy is a mechanism in which only some ATGs help to form an autophagosome-like vacuole (Codogno et al., 2012; Huang and Brumell, 2014). In this case, a subset of ATG proteins can be recruited to an already-existing (and likely damaged) membrane that is different from a phagophore, e.g., a vacuole containing *Salmonella* or mycobacteria. LC3-associated phagocytosis (LAP) is the best-studied example of a non-canonical autophagy pathway (Cemma and Brumell, 2012; Huang and Brumell, 2014). Toll-like receptors (TLRs) initiate LAP, then ATG5, Beclin-1, and ATG7 are subsequently recruited to promote phagosome maturation, lysosomal fusion, and killing of phagocytosed bacteria (Sanjuan et al., 2007; Lam et al., 2013; Martinez et al., 2015).

### Interplay between invasive bacteria and the autophagy machinery

In the case of bacterial autophagy (also called xenophagy), autophagy receptors such as p62 (sequestosome 1) and NDP52 (nuclear dot protein, 52 kDa) act as cytosolic sensors, bind ubiquitinated substrates and LC3 family proteins to selectively target bacteria to degradation by canonical autophagy (Levine et al., 2011). Canonical autophagy can control the fate of some intracellular bacteria, such as *Listeria monocytogenes* (Yoshikawa et al., 2009), *Francisella tularensis* (Checroun et al., 2006), *Salmonella enterica* subsp. *enterica* serovar Typhimurum (Thurston et al., 2009; Zheng et al., 2009), and *Mycobacterium tuberculosis* (Gutierrez et al., 2004). As a result, canonical autophagy is recognized as a critical component of innate immunity (Levine et al., 2011; Shibutani et al., 2015). On the other hand, some bacteria can replicate inside autophagosome-like structures generated by non-canonical autophagy (Huang and Brumell, 2014). *Legionella pneumophila* (Choy et al., 2012; Asrat et al., 2014), *Coxiella burnettii* (Newton et al., 2014), *Yersinia pseudotuberculosis* (Moreau et al., 2010), *Brucella abortus* (Starr et al., 2012), *Staphylococcus aureus* (Fraunholz and Sinha, 2012), and *Salmonella Typhimurium* (Yu et al., 2014) have been described to benefit from non-canonical autophagy.

To determine the precise role of autophagy in host defense against bacteria, it has been useful to investigate the role of bacterial autophagy *in vivo*. Animal models used to study bacterial autophagy *in vivo* include *Dictyostelium discoideum* (amoeba; Tung et al., 2010), *Caenorhabditis elegans* (nematode; Jia et al., 2009; Zou et al., 2014), *Drosophila melanogaster* (fruit fly; Yano et al., 2008), *Danio rerio* (zebrafish; Mostowy et al., 2013; van der Vaart et al., 2014), and *Mus musculus* (mouse; Castillo et al., 2012; Wang et al., 2012; Watson et al., 2012; Benjamin et al., 2013; Bonilla et al., 2013; Conway et al., 2013; Marchiando et al., 2013; Kimmey et al., 2015). In agreement with results obtained *in vitro*, the impact of autophagy *in vivo* depends on the bacterial pathogen under investigation. These alternative outcomes highlight the molecular complexity of bacterial autophagy. They also suggest difficulties in therapeutic manipulation of autophagy to protect against bacterial infection.

## Interactions between *S. flexneri* and the autophagy machinery

*Shigella* spp. are Gram-negative enteroinvasive pathogens, causing 163 million illness episodes worldwide per annum (Lima et al., 2015). *S. flexneri* possess a virulence plasmid which encodes a type III secretion system (T3SS), a needle-like apparatus used to inject bacterial effector proteins into the host cell and enable an intracellular lifestyle (Phalipon and Sansonetti, 2007; Ogawa et al., 2008). Minutes after invasion of host cells, including epithelial cells and macrophages, *S. flexneri* lyses the phagocytic vacuole and gains access to the host cytosol where it replicates (Ray et al., 2009; Fredlund and Enninga, 2014). To counteract *Shigella* replication in the cytosol, the host cell employs a variety of antimicrobial responses, including antibacterial autophagy and septin caging (septin caging as a mechanism of host defense will be described later in the text; Ogawa et al., 2005; Mostowy et al., 2010). However, to evade cytosolic immune responses, some cytosolic *Shigella* can subvert the host actin cytoskeleton to form propulsive actin tails and spread from cell-to-cell (Bernardini et al., 1989; Welch and Way, 2013). Thus, in addition to being an important human pathogen, *S. flexneri* has emerged as a paradigm to study cell-autonomous immunity and host cell biology during infection (Sansonetti, 2006; Ashida et al., 2011, 2015; Mostowy and Shenoy, 2015).

### Autophagy pathways induced during *Shigella* invasion of host cells

**Figure 1:**
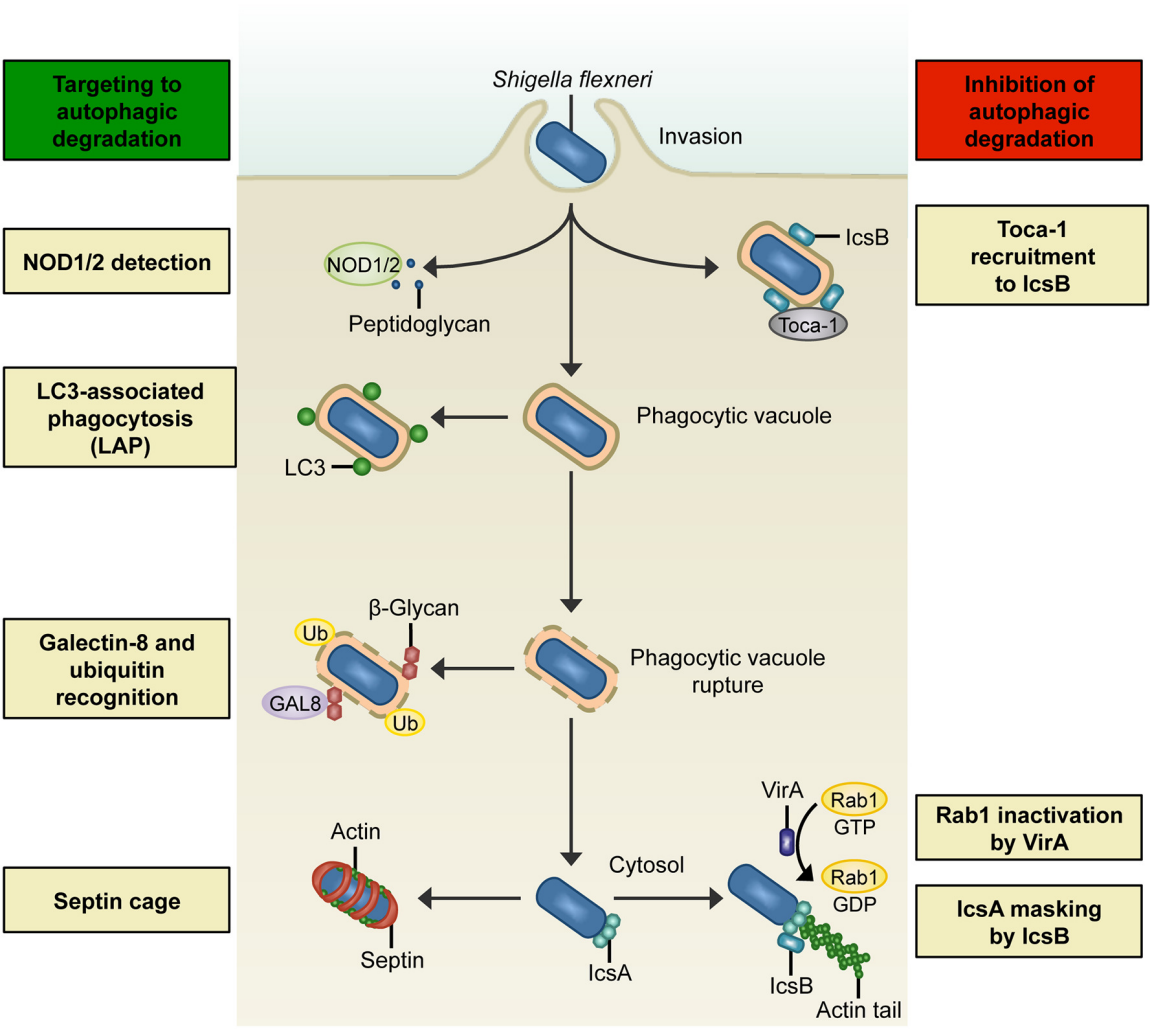
**Interactions between ***S. flexneri*** and the autophagy machinery. Left:** Summary of host mechanisms targeting *S. flexneri* to degradation by autophagic processes. NOD1/2 detects bacterial peptidoglycan during bacterial entry and recruits ATG16L, thereby triggering an autophagic response. In LC3-associated phagocytosis (LAP), a subset of autophagy proteins (e.g., LC3) is recruited to phagosomal membranes and promotes fusion with lysosomes. Membrane remnants can recruit autophagy components by ubiquitination (Ub), and also by recognition of host cell β-glycans by galectin-8 (GAL8). In the cytosol, actin-polymerizing bacteria can be recognized by ATG5 and entrapped in septin cage-like structures, thereby targeting bacteria to autophagic degradation and preventing their dissemination. **Right:** Overview of *S. flexneri* effectors that inhibit degradation by autophagic processes. During invasion, the bacterial effector IcsB recruits Toca-1, which prevents the recruitment of LC3 and other autophagy markers. In the cytosol, *Shigella* can circumvent Atg5-recognition of IcsA and septin caging by expressing IcsB. Another mechanism of *S. flexneri* to inhibit autophagosome formation in the cytosol is to secrete VirA to inactivate Rab1.

*S. flexneri* invasion of normally non-phagocytic epithelial cells relies upon T3SS effector proteins to induce reorganization of the host cell cytoskeleton, membrane ruffling, and bacterial uptake (Cossart and Sansonetti, 2004). Following entry, the pattern recognition nucleotide-binding oligomerization domain (NOD)- like receptors NOD1 and NOD2 detect bacterial peptidoglycan and trigger pro-inflammatory signaling cascades that restrict bacterial survival (Philpott et al., 2014). Work using the human epithelial cell line HeLa and *S. flexneri* has demonstrated that NOD proteins interact with ATG16L1, thereby recruiting the autophagy machinery to the bacterial entry site at the plasma membrane (Travassos et al., 2010). These data suggest that NOD proteins link bacterial sensing with autophagosome biogenesis (Figure 1). Interestingly, ATG16L1 also has an autophagy-independent role in the control of *Shigella* and NOD-mediated inflammatory responses in epithelial cells (Sorbara et al., 2013). In this case, ATG16L1 inhibits NOD1- and NOD2-driven cytokine responses to cytosolic bacteria.

LAP can also occur during *Shigella* invasion of epithelial cells (Figure 1), where LAP is dependent on the activity of the T3SS (Campbell-Valois et al., 2015). However, *Shigella* has a mechanism mediated by the T3SS effector IcsB to counteract LAP. IcsB is a 52 kDa protein that uses the bacterial chaperone IpgA for its stability (Ogawa et al., 2003). Secreted IcsB localizes around the bacterial surface, and though not required for bacterial entry, plays an important role during cell-to-cell spread. Soon after bacterial entry, IcsB recruits transducer of CDC42-dependent actin assembly 1 (Toca-1) to prevent the recruitment of NDP52 and LC3 (Baxt and Goldberg, 2014). In uninfected cells, Toca-1 and neuronal Wiskott-Aldrich syndrome protein (N-WASP) are recruited to the membrane by direct interactions with the Rho guanosine triphosphatase (GTPase) CDC42 (Ho et al., 2004). In contrast, *S. flexneri* recruits Toca-1 via IcsB (to inhibit recognition by autophagy machinery) and N-WASP via IcsA (to polymerize actin; Leung et al., 2008; Baxt and Goldberg, 2014). Of note, *S. flexneri* can also be recognized by LC3 after cell-to-cell spreading, when the bacterium is entrapped in a double-membrane vacuole (Campbell-Valois et al., 2015). Here, *Shigella* can avoid autophagic degradation by secreting IcsB and VirA (the virulence factor VirA will be described later in the text), which act synergistically to promote bacterial escape from LC3-positive vacuoles.

Together, different sensing mechanisms including NOD1/2 and LAP trigger autophagic recognition of invading *Shigella*. These mechanisms can be viewed as a crucial aspect of host defense to control bacteria at the onset of bacterial interactions with the host cell.

### Autophagy pathways induced by *Shigella* rupture of the phagocytic vacuole

Shortly after invasion of host cells, *S. flexneri* can exit the phagocytic vacuole and gain access to the cytosol. In epithelial cells, rupture of the phagocytic vacuole by *Shigella* can initiate autophagy by inducing nutrient starvation (Tattoli et al., 2012). In this case, *Shigella* causes downregulation of the autophagy inhibitor mammalian target of rapamycin complex 1 (mTORC1, which inhibits the ULK1–Atg13–FIP200 complex in the presence of nutrients). From this, it has been proposed that *Shigella*-induced amino acid starvation can be sensed by the host cell to initiate an immune response.

When *Shigella* ruptures its phagocytic vacuole in epithelial cells, membrane remnants can be ubiquitinated (Dupont et al., 2009; Figure 1). Here, autophagy receptors p62 and NDP52 recognize ubiquitinated membrane remnants and recruit LC3-positive membrane for autophagosome biogenesis. Moreover, p62 on membrane remnants colocalizes with nuclear factor kappa B (NF-κB) signaling molecules, including tumor necrosis factor (TNF) receptor-associated factor 6 (TRAF6), thereby serving to dampen the inflammatory response (Dupont et al., 2009). Thus, autophagic degradation has important roles in the control of both infection and inflammation (Shi et al., 2012; Deretic et al., 2013; Shibutani et al., 2015).

Membrane damage exposes host cell glycans to the cytosol, which are damage-associated molecular patterns (DAMPs) recognized by galectins, a family of beta-galactoside-binding proteins (Figure 1). Studies using epithelial cells and macrophages have demonstrated that galectin-3 and galectin-8 accumulate at sites of membrane damage induced by *Shigella* (Paz et al., 2010; Thurston et al., 2012). While a role for galectin-3 in host defense is not yet clear, galectin-8 initiates the recruitment of NDP52 and LC3 to direct the autophagy machinery to the ruptured phagocytic vacuole (Thurston et al., 2012). The galectin-8-NDP52-LC3 pathway is viewed to occur upstream of the ubiquitin-NDP52-LC3 pathway also targeting the phagocytic vacuole ruptured by *Shigella* (Paz et al., 2010; Thurston et al., 2012; Boyle and Randow, 2013). In both cases, membrane can act as a danger signal used to direct autophagy components toward invasive bacteria. However, canonical autophagy may also promote repair of damaged vacuolar membranes, as in the case of *S. Typhimurium*-containing vacuoles (Kreibich et al., 2015). Therefore, the precise role of membrane recognition by the autophagy machinery in the restriction of bacterial proliferation is unknown.

### Autophagy pathways induced by *Shigella* in the cytosol

Once in the cytosol, *S. flexneri* can subvert the host actin cytoskeleton by expressing the outer membrane autotransporter protein IcsA to recruit N-WASP and the actin related protein 2/3 (ARP2/3) complex, inducing actin-based motility to spread from cell-to-cell (Welch and Way, 2013). IcsA-mediated actin polymerization is also required for recognition of cytosolic *Shigella* by the autophagy machinery in epithelial cell lines and mouse embryonic fibroblasts (MEFs; Ogawa et al., 2005; Mostowy et al., 2010, 2011). IcsA is recognized by ATG5, triggering the formation of an isolation membrane (Ogawa et al., 2005). Infection of epithelial cell lines and MEFs has shown that this process can be independent of ubiquitin, and is coincident with ATG5 binding to the adaptor protein tectonin beta-propeller repeat containing 1 (TECPR1; Ogawa et al., 2011). TECPR1 interacts with WIPI-2 and PI(3)P, and recruits LC3 to target *Shigella* to autophagic degradation.

To protect itself from autophagic recognition, cytosolic *S. flexneri* can inhibit the interaction between IcsA and ATG5 by secreting IcsB, which binds to IcsA and competitively inhibits ATG5 binding (Ogawa et al., 2005; Figure 1). The region of IcsA bound by IcsB/ATG5 overlaps with one of the three N-WASP interacting regions (Ogawa et al., 2005; May and Morona, 2008). It is unknown if N-WASP competes with IcsB/ATG5 in binding to the same region of IcsA, or whether the two other binding regions of IcsA are sufficient to recruit N-WASP. Bacteria without IcsB exhibit an intracellular replication defect in MEFs, highlighting the crucial role of ATG5 recognition in the control of *Shigella* (Ogawa et al., 2005). Historically, this discovery made *Shigella* the first example of an intracellular pathogen having an effector that inhibits autophagic recognition for intracellular survival.

*Shigella* expression of VirA is a separate mechanism to counteract autophagy in epithelial cells (Dong et al., 2012). VirA is localized upstream of IcsA on the *Shigella* virulence plasmid and is secreted during invasion of host cells (Uchiya et al., 1995), where it acts as a GTPase-activating protein (GAP) that inactivates the GTPase Rab1 (Dong et al., 2012; Figure 1). Rab1, implicated in vesicular transport between the endoplasmic reticulum (ER) and the Golgi, has an important role in autophagosome formation (Zoppino et al., 2010). By inactivating Rab1, VirA disrupts ER-to-Golgi trafficking, and mediates suppression of autophagosome formation against *S. flexneri* (Dong et al., 2012). VirA is one of 2 bacterial effectors discovered so far, the other being the type 4 secretion system (T4SS) effector RavZ from *L. pneumophila* (Choy et al., 2012), that can directly interfere with the autophagy machinery (Huang and Brumell, 2014; Mostowy and Shenoy, 2015).

Septins, GTP-binding proteins essential for cell division (Saarikangas and Barral, 2011; Mostowy and Cossart, 2012b), interact with the autophagy machinery and play a crucial role in the control of cytosolic *S. flexneri* (Mostowy et al., 2010, 2011). Septins are recognized as cytoskeletal components because they form filaments that associate with cellular membranes, actin filaments, and microtubules (Saarikangas and Barral, 2011; Mostowy and Cossart, 2012b; Bezanilla et al., 2015). In epithelial cells, septins are recruited to sites of IcsA-mediated actin polymerization and form cage-like structures necessary for the recruitment of ubiquitin, p62, NDP52, and LC3 to cytosolic *Shigella* (Mostowy et al., 2010, 2011; Figure 1). Consistent with its role as an autophagy inhibitor, IcsB also masks *Shigella* from septin caging (Mostowy et al., 2010, 2011). Importantly, septin cages have also been observed *in vivo* using zebrafish, highlighting septin assembly as an evolutionarily conserved determinant of host defense (Mostowy et al., 2013). From the *Shigella*-zebrafish infection model, p62 is crucial for septin caging and the restriction of bacterial replication by autophagy *in vivo*. On the other hand, rapamycin (an inhibitor of mTOR and stimulant of autophagy) can increase *Shigella* replication and decrease zebrafish survival. Overall, these data indicate that host survival depends on the appropriate autophagic response to control *Shigella* infection *in vivo* (Mostowy et al., 2013). In the case of humans, the ATG16L1 T300A polymorphism linked to Crohn's disease, an inflammatory bowel disease, can reduce the process of selective autophagy and host defense against *Shigella* (Lassen et al., 2014).

## Discussion

Originally discovered as a bulk degradation process important for cellular homeostasis (Yang and Klionsky, 2010), autophagy has since been recognized as important for cell-autonomous immunity and host defense (Levine et al., 2011; Mostowy and Shenoy, 2015; Shibutani et al., 2015). On the other hand, some intracellular pathogens have evolved mechanisms to evade or interfere with autophagy processes for intracellular survival (Mostowy and Cossart, 2012a; Choy and Roy, 2013; Huang and Brumell, 2014). Adding to this complexity, it is now clear that ATG proteins also have autophagy-independent roles in immunity and cellular homeostasis (Huang and Brumell, 2014; Mostowy and Shenoy, 2015). In this review, we have highlighted how studies using *Shigella* can help to investigate the breadth of autophagy responses available to the host.

The *S. flexneri* infection model can be used to study key issues in cell-autonomous immunity and host cell biology, including the ability of host cells to sense and defend against intracellular bacteria. The host cell has evolved multiple sensing mechanisms that together enable a multi-tiered defense network to counteract *Shigella* invasion (Table 1). However, *Shigella* has co-evolved a variety of protection mechanisms, and in some cases can interfere with host cell biology to circumvent autophagic processes (Table 1). Despite the understanding gained from using the *Shigella* infection model, many outstanding issues remain. For example, what is the role of the host cytoskeleton in autophagy and its ability to restrict or promote bacterial replication? Actin, microtubules, intermediate filaments, and septins are four main cytoskeletal components of mammalian cells, yet their precise roles in autophagy are not understood (Mostowy, 2014). What is the link between autophagy of bacteria and autophagy of mitochondria (also called mitophagy)? Mitochondria can be viewed as bacteria-derived endosymbionts, and recent studies have discovered that autophagy of bacteria and mitochondria use the same machinery (Manzanillo et al., 2013; Randow and Youle, 2014). Finally, to fully determine the role of autophagy in host defense against *Shigella* will require in depth experimentation to be performed *in vivo*. Though mammalian models remain poorly suited to image the cell biology of *Shigella* infection *in vivo*, the natural translucency of zebrafish larvae enables non-invasive *in vivo* imaging at high resolution throughout the organism (Kanther and Rawls, 2010; Renshaw and Trede, 2012). Exploiting this, the *in vivo* role of *Shigella* interactions with the autophagy machinery can be examined at the subcellular, cellular, and whole-animal level (Mostowy et al., 2013).

**Table 1:** *****S. flexneri*** mechanisms to induce, evade, or interfere with the autophagy machinery**.

**Bacterial effector**	**Role**	**References**
**INDUCER OF AUTOPHAGOSOME FORMATION**		
Peptidoglycan	Recognized by NOD1/2 and recruits ATG16L1 to entry site	Travassos et al., 2010; Sorbara et al., 2013
Active T3SS	Required for LC3 recruitment during LAP	Campbell-Valois et al., 2015
IcsA	Recognized by ATG5 inducing TECPR1 recruitment and septin caging	Ogawa et al., 2005, 2011; Mostowy et al., 2010, 2011
**EVASION OF AUTOPHAGY RECOGNITION**		
IcsB (early)	Interacts with Toca-1 to prevent recognition by NDP52	Baxt and Goldberg, 2014
IcsB (late)	Prevents ATG5 recognition of IcsA, counteracting recruitment of ubiquitin, p62, NDP52, and TECPR1, and septin caging	Ogawa et al., 2005, 2011; Mostowy et al., 2010, 2011
IcsB, VirA	Promotes escape from LC3-positive vacuoles after cell-to-cell spread	Campbell-Valois et al., 2015
**INTERFERENCE WITH AUTOPHAGY MACHINERY**		
VirA	Inactivates Rab1 to inhibit autophagosome formation	Dong et al., 2012

Taken together, outcomes generated from studying the interplay between *S. flexneri* and the autophagy machinery *in vitro* using tissue culture cells and *in vivo* using animal models can help to better understand fundamental mechanisms of host defense. They could additionally suggest the development of novel therapies for infectious diseases.

## Author contributions

All authors listed, have made substantial, direct and intellectual contribution to the work, and approved it for publication.

### Conflict of interest statement

The authors declare that the research was conducted in the absence of any commercial or financial relationships that could be construed as a potential conflict of interest.
